# Poststroke emotional disturbances and a tryptophan hydroxylase 2 gene polymorphism

**DOI:** 10.1002/brb3.892

**Published:** 2018-01-11

**Authors:** Mihye Ko, Smi Choi‐Kwon, Sang‐Eun Jun, Ju Han Kim, Kyung‐Hee Cho, Hyun‐Wook Nah, Hasup Song, Jong S. Kim

**Affiliations:** ^1^ College of Nursing The Research Institute of Nursing Science Seoul National University Seoul South Korea; ^2^ College of Nursing Keimyung University Daegu South Korea; ^3^ College of Medicine Seoul National University Seoul South Korea; ^4^ Department of Neurology Korea University Seoul South Korea; ^5^ Dong‐A University Busan South Korea; ^6^ University of Ulsan Asan Medical Center Seoul South Korea

**Keywords:** depression, emotional disturbances, gene, polymorphism, stroke, tryptophan hydroxylase

## Abstract

**Objectives:**

Emotional dysfunction is a common finding in stroke patients. Despite reports on serotonergic involvement in the etiology of poststroke emotional dysfunction (PSED), the role of serotonin synthesizing tryptophan hydroxylase 2 (TPH2) genes in the development of PSED remains unclear.

**Methods:**

Genotyping of *TPH2* rs4641528 and rs10879355 was performed from genomic DNA of 383 stroke patients collected previously and stored at −70°C. Potential associations between *TPH*2 genes and poststroke depression (PSD), poststroke emotional incontinence (PSEI), and poststroke anger proneness (PSAP) were investigated 3 months poststroke.

**Results:**

Among the 383 patients, 69 (18%) had PSD, 41 (11%) had PSEI, and 93 (24%) had PSAP. The *TPH2* rs4641528 genotype frequencies differed significantly between patients with and without either PSD or PSEI, although no significant differences were found between the patients with and without PSAP. In multiple logistic regression analysis, PSD was related to the National Institutes of Health Stroke Scale (NIHSS) score at admission (95% confidence interval [CI]: 1.047–1.230, *p *<* *.01), modified Rankin scale score at 3 months (95% CI: 0.135–0.848, *p *<* *.05), and *TPH2* rs4641528 C allele (95% CI: 1.039–5.631, *p *<* *.05), whereas PSEI was associated only with the NIHSS score at admission (95% CI: 1.053–1.259, *p *<* *.01) and the *TPH2* rs4641528 C allele (95% CI: 1.029–11.678, *p *<* *.05).

**Conclusions:**

Our findings suggest that the *TPH2* rs4641528 C allele may play a role in the pathogenesis of PSD and PSEI but not PSAP in Korean stroke patients.

## 1 INTRODUCTION

Emotional dysfunction, which includes depression, emotional incontinence, and anger proneness, is commonly observed in stroke patients (Kim & Choi‐Kwon, 2000; Kim, Choi, Kwon, & Seo, 2002; Choi‐Kwon et al., 2012; Hackett, Yang, Anderson, Horrocks, & House, 2010). Although the causes of poststroke emotional dysfunction (PSED) are complex and multifactorial, disturbances in the central serotonin system have been considered to play an important role (Hackett et al., 2010). An association between the serotonin transporter (5‐HTT) gene and PSED development has been demonstrated in Western countries (Moller, Andersen, & Gjedde, 2007) and in Korea (Choi‐Kwon et al., 2012; Baud et al., 2009). In a recent meta‐analysis, homozygous short variation in the serotonin transporter‐linked polymorphic region (5‐HTTLPR) was found to be a risk factor for PSED (Mak, Kong, Mak, Sharma, & Ho, 2013).

Further, we postulated that the tryptophan hydroxylase 2 (TPH2) genes responsible for serotonin synthesis may be associated with PSED. TPH2 genes are expressed in brain regions such as the frontal cortex, thalamus, hippocampus, hypothalamus, and amygdala, and more predominantly in the brain stem, the major locus of the serotonin‐producing neurons (Zill et al., 2007).

The aim of this study, therefore, was to investigate whether *TPH2* gene polymorphisms were associated with PSED.

## 2 SUBJECTS AND METHODS

This is a secondary data analysis from a parent study (Choi‐Kwon et al., 2012; Choi‐Kwon et al., 2013). The design and methods of the study were published previously (Choi‐Kwon et al., 2012) and are briefly summarized here.

### 2.1 Procedures

Participants were recruited from consecutive patients who were admitted to the Asan Medical Center between March 2008 and February 2010 with acute ischemic stroke as confirmed by diffusion‐weighted magnetic resonance imaging (DWI) correlated with neurological symptoms within 72 hr after onset. Excluded were patients who: (1) had intracerebral hemorrhage, subarachnoid hemorrhage, arteriovenous malformation, venous infarction, or moyamoya disease; (2) had a transient ischemic attack without DWI‐identified lesion; (3) had communication problems (aphasia, dementia, or dysarthria) severe enough as to be unable to undergo a reliable interview; (4) scored ≤23 on the Mini‐Mental State Examination or had a history of diagnosed depression or other psychiatric illnesses before the onset of stroke; (5) had been taking serotonin reuptake inhibitors (SSRIs) for any reason; (6) lived alone so that information from relatives was not available; or (7) did not give consent.

The patients’ neurological findings including NIHSS were recorded by one of three stroke neurologists (K.H.C., H.W.N., and H.S.). The locations of the lesions detected by DWI were analyzed by one of the authors (J.S.K.) and categorized as previously described (Choi‐Kwon et al., 2012).

Because mood and emotional symptoms fluctuate in the acute stages associated with changing neurological deficits, we conducted outpatient interviews in the subacute stage (3–4 months after stroke onset) once patients’ neurological status had stabilized. Poststroke depression (PSD) and poststroke emotional incontinence (PSEI) were assessed using the Beck Depression Inventory and Kim's criteria, respectively, as previously reported (Choi‐Kwon et al., 2012). Poststroke anger proneness (PSAP) was assessed using the Spielberger trait anger scale (Spielberger, Jacobs, Russell, & Crane, 1983). For statistical purposes, each patient's score on the modified Rankin scale (mRS) was recorded and categorized as severe (3–6) or mild (0–2).

The institutional review board at Asan Medical Center approved the study. All participants provided written informed consent.

### 2.2 Genotyping

Blood samples were collected at admission, and two of 13 tag SNPs (rs4641528, rs10879355) from the Korean population (KOR) HapMap Database (Hapmap Database 3/Rel #2, Feb 09, Minor Allele Frequencies (MAFs) of more than 0.1) were selected for evaluation. These two SNPs were chosen as they maximize SNP prediction accuracy, hence sufficiently represent other tag SNPs (Stram, 2004; Halperin, Kimmel, & Shamir, 2005). Mean max *r* ^2^ of the two SNPs is 0.953 using the “Tagger” program, an implement of the Haploview software (Mouri et al., 2009; Park et al., 2010).

We used genomic DNA that was isolated and stored in a −70°C freezer using a QIAamp DNA mini kit (QIAGEN Inc., Hilden, Germany). We routinely monitored extracted DNA samples with spectrophotometer (Nano drop 1000) for quality assurance. The polymorphic regions were amplified by a polymerase chain reaction (PCR) (Thermal Cycler 2720; Applied Biosystems, Foster City, CA, USA) with 100 ng of DNA and 10 μm of each primer of *TPH2* genes with the following sequences: rs4641528, forward 5ʹ‐ AAA GAT ACC TGT CTT TTG CTC ACT TT‐3ʹ and reverse 5ʹ‐CTG GCT CCA AAT GAA AGC TCA GGC A‐3ʹ; and rs10879355, forward 5ʹ‐CCC AAG TCA TAC TCG TGT TCA CTC AC‐3ʹ and reverse 5ʹ‐ACT CTT GTG TGC CAC TGC CAT CTA G‐3ʹ (Nielsen & Rehfeld, 1994; Yoon, Yang, Lee, & Kim, 2008). We consulted Bionics.co (Seoul, Korea) for genotyping of the TPH2 polymorphisms. Final PCR products with a value <99% were excluded from the analysis.

For rs4641528 and rs10879355, the amplification mixture contained 1.25‐μl *Taq* polymerase (TaKara, Japan), 1‐μl genomic DNA, 5‐μl 10× PCR buffer, 1‐μl dNTP mixture, 2 μl of each primer, and 27.75‐μl distilled water. The thermal cycling conditions were as follows: 95°C for 15 min, followed by 35 cycles of 95°C for 15 s, 62°C for 20 s, 72°C for 20 s, and a final extension step at 72°C for 10 min.

### 2.3 Analysis

The presence of the Hardy–Weinberg equilibrium was tested by the χ^2^ test for goodness of fit. Group differences were analyzed using descriptive statistics, including the Student's *t* test, χ^2^ test, and Fisher's exact test (SPSS version 11.5). Linkage disequilibrium between the *TPH2* SNPs was determined using Haploview software program version 3.32 (http://www.broad.mit.edu/mpg/haploview/). Multiple logistic regression analysis was used to determine whether PSD, PSEI, or PSAP was influenced by the *TPH2* gene variants after controlling for age, sex, and factors related to PSD, PSEI, and PSAP as covariates. Statistical significance was defined at *p *<* *.05.

## 3 RESULTS

Of 508 patients who initially agreed to be involved in this study at stroke onset, 86 were lost because of inadvertent sampling errors (insufficient volume of DNA), and 39 were excluded because they could not be reached 3 months poststroke for the following reasons: physical (7, 18%) or cognitive deterioration (5, 12.8%), refusal to give consent (10, 25.6%) death (5, 12.8%), or insufficient follow‐up due to relocation (12, 30.7%). No differences were found in age, sex, educational level, or National Institutes of Health Stroke Scale (NIHSS) score at admission between the patients who were included (*n *=* *383) and those who were not (*n *=* *86).

Patient characteristics are listed in Table 1. Among the 383 patients, 69 (18%) had PSD, 41 (11%) had PSEI, and 93 (24%) had PSAP at 3 months following stroke (Table 1). PSD was associated with education level (*p *<* *.05), high NIHSS score at admission (*p *<* *.01), and mRS score after 3 months (*p *<* *.01); PSEI was associated with lesion location (*p *<* *.05), high NIHSS score at admission (*p *<* *.01), and mRS score at 3 months (*p *<* *.05); and PSAP was not associated with any of the factors.

**Table 1:** Factors associated with PSD, PSEI, and PSAP 3 months after stroke

Variable	PSD		PSEI		PSAP	
	Present (*n* = 69)	Absent (*n* = 314)	Present (*n* = 41)	Absent (*n* = 342)	Present (*n* = 93)	Absent (*n* = 290)
Age, year, mean ± *SD*	62.9 ± 11.7	61.0 ± 12.5	60.0 ± 10.0	61.6 ± 12.6	62.5 ± 11.6	61.1 ± 12.6
Male, *n* (%)	37 (53.6)	203 (64.6)	26 (63.4)	214 (62.6)	57 (61.3)	183 (63.1)
Education, years, mean ± *SD*	9.0 ± 5.1**	10.5 ± 4.8	10.8 ± 4.7	10.2 ± 4.9	10.1 ± 5.2	10.3 ± 4.8
Previous stroke: *n* (%)	10 (14.5)	57 (18.2)	7 (17.1)	60 (17.5)	17 (18.3)	50 (17.2)
Lesion location						
Anterior cortex	11 (15.9)	53 (16.9)	7 (17.1)	57 (16.7)	10 (10.8)	54 (18.6)
CR + BG + IC	28 (40.6)	86 (27.4)	15 (36.6)	99 (28.9)	29 (31.2)	85 (29.3)
Thalamus	4 (5.8)	28 (8.9)	1 (2.4)	31 (9.1)	13 (14.0)	19 (6.6)
Medulla	6 (8.7)	20 (6.4)	2 (4.9)	24 (7.0)	6 (6.5)	20 (6.9)
Pons + midbrain	7 (10.1)	51 (16.2)	12 (29.30)	46 (13.5)	17(18.3)	41 (14.1)
Cerebellum	6 (8.7)	32 (10.2)	1 (2.4)	37 (10.8)	6 (6.5)	32 (11.0)
Posterior cortex	7 (10.1)	44 (14.0)	3 (7.3)	48 (14.0)	12 (12.9)	39 (13.4)
Laterality, *n* (%)						
Right	38 (55.1)	158 (50.3)	25 (61.0)	171 (50.0)	53 (57.0)	143 (49.3)
Left	30 (43.5)	142 (45.2)	16 (39.0)	156 (45.6)	37 (39.8)	135 (46.6)
Bilateral	1 (1.4)	14 (4.5)	0 (0.0)	15 (4.4)	3 (3.2)	12 (4.1)
NIHSS score at admission ± *SD*	5.5 ± 3.3*	3.6 ± 3.2	5.8 ± 3.7*	3.7 ± 3.2	4.2 ± 3.7	3.8 ± 3.2
mRS score at 3 months: severe, *n* (%)						
Severe	12 (17.4)*	13 (4.1)	7 (17.1)**	18 (5.3)	4 (4.3)	21 (7.2)
Mild	57 (82.6)	301 (95.9)	34 (82.9)	324 (94.7)	89 (95.7)	269 (92.8)
rs4641528						
C (C/C+C/T)	62 (89.9)*	238 (75.8)	38 (92.7)**	262 (76.6)	75 (80.6)	225 (77.6)
T/T	7 (10.1)	76 (24.2)	3 (7.3)	80 (23.4)	18 (19.4)	65 (22.4)
rs10879355						
C (C/C+C/T)	53 (76.8)	247 (78.7)	33 (80.5)	267 (78.1)	69 (74.2)	231 (79.7)
T/T	16 (23.2)	67 (21.3)	8 (19.5)	75 (21.9)	24 (25.8)	59 (20.3)

BG, basal ganglia; CR, corona radiata; IC, internal capsule; mRS, modified Rankin scale; NIHSS, National Institutes of Health Stroke scale; PSD, poststroke depression; PSEI, poststroke emotional incontinence; PSAP, poststroke anger proneness.

*p <* *.05, **p <* *.01.

The genotype frequencies of the *TPH2* SNP rs4641528 differed significantly between the patients with and without PSD or PSEI, and the TT homozygote of rs4641528 was less common in patients with these conditions than in those without (*p *=* *.01 and *p *=* *.05, respectively). No significant differences were found in the *TPH2* SNPs between the patients with PSAP and those without (Table 1).

Table 2 presents the results of multiple logistic backward stepwise regression analysis for PSD and PSEI. NIHSS at admission (95% confidence interval [CI]: 1.047–1.230, *p *<* *.01), mRS at 3 months (95% CI: 0.135–0.848, *p *<* *.05), and *TPH2* rs4641528 C allele carriers (CC+CT genotype) (95% CI: 1.039–5.631, *p *<* *.05) were related to PSD whereas PSEI was associated with NIHSS score at admission (95% CI: 1.053–1.259, *p *<* *.01) and with *TPH2* rs4641528 C allele carriers (CC+CT genotype) (95% CI: 1.029–11.678, *p *<* *.05) (Table 2).

**Table 2:** Factors associated with PSD and PSEI 3 months after stroke

Variable	B	*SE*	*p*	Exp(B)	95% CI
PSD F/U at 3 months					
Education years ± *SD*	−0.052	0.029	.069	0.949	0.897–1.004
NIHSS score at admission ± *SD*	0.127	0.041	.002	1.135	1.047–1.230
mRS score at 3 months	−1.083	0.468	.021	0.339	0.135–0.848
rs4641528 (C/C+C/T)	0.883	0.431	.040	2.419	1.039–5.631
Constant	−1.362	0.691	.049	0.256	
PSEI F/U at 3 months					
NIHSS score at admission ± *SD*	0.141	0.046	.002	1.151	1.053–1.259
mRS score at 3 months	−0.888	0.507	.080	0.412	0.152–1.111
rs4641528 (C/C+C/T)	1.243	0.620	.045	3.466	1.029–11.678
Constant	−3.043	0.817	.000	0.048	

F/U, follow‐up; mRS, modified Rankin scale; NIHSS, National Institutes of Health Stroke scale; PSD, poststroke depression; PSEI, poststroke emotional incontinence.

## 4 DISCUSSION

In this study, we investigated for the first time the association between the *TPH2* SNPs and PSED, including PSD, PSEI, and PSAP. The *TPH2* SNPs (rs4641528 and rs10879355) in our patients showed similar genotype distributions to the HapMap CEU data set.

We found that carrying the C allele of rs4641528 in the *TPH2* gene was associated with PSD and PSEI 3 months poststroke. Our findings are consistent with the previous results that PSD and PSEI are associated with lesion locations where serotonergic fibers are abundant (Kim & Choi‐Kwon, 2000) and that *TPH2* mRNA is abundant in the brainstem, the main locus of the serotonin‐producing neurons (Zhang, Beaulieu, Sotnikova, Gainetdinov, & Caron, 2004). Moreover, studies have reported a positive association between the *TPH2* gene and depression in patients with personality disorders or depression (Perez‐ Rodriguez et al., 2010; de Araujo Pereira et al., 2011; Gao et al., 2012).

*TPH2* rs4641528 has been reported to have high linkage disequilibrium (≥0.8) with other *TPH2* SNPs related to affective disorders: rs7305115 with late‐onset depression in Brazilian (de Araujo Pereira et al., 2011) and Chinese populations (Wang et al., 2015); rs2171363 (Tsai et al., 2009) and rs10879346 (Tzvetkov, Brockmoller, Roots, & Kirchheiner, 2008) with major depression and response to antidepressant treatment; rs1352250 to hopelessness, a potential endophenotype for suicidal behavior (Lazary et al., 2012); and rs12229394 with depression accompanied by fatigue (Utge et al., 2010). Therefore, it is likely that *TPH2* rs4641528 is an important SNP representing other SNPs related to emotional dysfunction not just in stroke patients but also in other psychiatric patients in different ethnic populations.

Positive relationships between PSED and 5‐HT transporter genes *5‐HTTLPR* (ss genotype) and *STin2* variable number tandem repeat (VNTR) have been reported previously (Choi‐Kwon et al., 2012; Mak et al., 2013; Kohen et al., 2008; Ramasubbu, Tobias, & Bech‐Hansen, 2008; Fang, Yan, Jiang, Li, & Cheng, 2011). Our data further support the hypothesis that there is a relationship between PSED and serotonin gene polymorphism, particularly in serotonin synthesis (Choi‐Kwon et al., 2012).

We found that the NIHSS score at admission and the mRS score 3 months after stroke were also related to PSD and PSEI in multiple logistic regression analysis. Because these items are closely related, we performed further analysis after excluding either the NIHSS score at admission or the mRS score at 3 months. The results were identical, supporting our finding that these two factors independently affect PSD and PSEI in the subacute stages (data not shown). These findings illustrate that PSD and PSEI may be caused by multiple factors such as neurological dysfunction and functional deficits in addition to genetic traits.

Because the *TPH2* gene is associated with depression in subjects without stroke (Gao et al., 2012) one may argue that our findings are not specific to PSD. It has been shown that PSD is associated with a complex etio‐pathogenesis that includes psychologic responses secondary to sudden functional disability, neurochemical changes due to brain damage, and difficult familial, social, or environmental situations (Kim, 2016). On top of these, certain genetic traits for depression may be related to the development of PSD, and our results may provide some evidence to support this argument. In our study, we excluded patients who had been diagnosed with depression prior to the stroke event. However, we suspect that a genetic predisposition for depression may be one factor that increases vulnerability to depression in these patients. Stated another way, PSD may become more apparent if patients harbor this genetic trait even if they are nondepressive before the onset of stroke. Alternatively, TPH2 may be associated with depression through other mechanisms. Elevation of glucocorticoid creates a vulnerability to emotional disturbance (Mak, Tang, Chan, Cheak, & Ho, 2011); and elevation of corticosterone is associated with increased TPH2 expression (Donner, Montoya, Lukkes, & Lowry, 2012). Increased TPH2 expression was associated with an increase in leptin and interleukin‐6 (IL‐6) (Reichardt et al., 2013) and a decrease in GABA (Waider et al., 2013); which play a key role in the pathogenesis of emotional disturbance (Liu, Ho, & Mak, 2012; Yang, De Xiang Liu, Pan, Ho, & Ho, 2016; Lu et al., 2017).

Finally, although the serotonin system has been reported to be associated with PSAP (Choi‐Kwon et al., 2006) in the current study, we found no significant association between *TPH2* genotyping and PSAP. The 5‐HT_1B_ receptor gene, which we did not evaluate, may play a role in mediating impulsivity or aggregation (Giegling, Hartmann, Moller, & Rujescu, 2006; Nomura & Nomura, 2006; Zouk et al., 2007). Alternatively, anger proneness may be closely associated with dopaminergic (Choi‐Kwon et al., 2013; Schluter et al., 2013) rather than serotonergic system dysfunction (Grimes, Ricci, & Melloni, 2007).

There are limitations in the present study. First, we investigated two SNPs (rs4641528 and rs10879355) in the *TPH2* gene. These SNPs can provide only a partial insight into the genetic basis of complex disorders such as PSED. Second, because many patients were excluded from this analysis, the prevalence of PSED in our study may not be generalized. Finally, this is a preliminary study and multiple comparison tests were not conducted although two SNPs were investigated. Despite these limitations, our results showed that PSD and PSEI are related to *TPH2* gene polymorphisms. Further studies are needed to confirm our preliminary findings and to develop strategies to identify and treat patients who are at risk of PSED.

## CONFLICT OF INTEREST

On behalf of all authors, the corresponding author states that there is no conflict of interest.
